# Plant Tissue Localization and Morphological Conversion of *Azospirillum brasilense* upon Initial Interaction with *Allium cepa* L.

**DOI:** 10.3390/microorganisms7090275

**Published:** 2019-08-21

**Authors:** Leidong Hong, Yoshitake Orikasa, Hisayo Sakamoto, Takuji Ohwada

**Affiliations:** 1 Department of Life and Food Science, Obihiro University of Agriculture and Veterinary Medicine, Inada-cho, Obihiro, Hokkaido 080-8555, Japan; 2 United Graduate School of Agricultural Science, Iwate University, 18-8 Ueda-sanchome, Morioka, Iwate 020-8550, Japan

**Keywords:** *Azospirillum brasilense*, *Allium cepa* L., plant tissue localization, morphological conversion, c-form

## Abstract

The genus *Azospirillum* is recognized as plant growth-promoting bacteria that exert beneficial effects on the host plant and is morphologically converted into cyst-like cells (i.e., c-form) in association with poly-β-hydroxybutyrate (PHB) accumulation in the cells under stress conditions. We constructed *Azospirillum brasilense*, labeled with reporter genes (*gus/gfp*, *mCherry*) and examined the plant tissue localization along with a morphological conversion into the c-form upon its initial interaction with onion seedlings (*Allium cepa* L.). The PHB granules in the *A. brasilense* cells were easily detected under fluorescence as “black holes”, rendering it possible to monitor the morphological conversion from vegetative to the c-form cells. The results showed that the *A. brasilense* cells on the surface of the roots and bulbs (underground stem) began converting at three days following inoculation and that the cell conversion was significantly advanced with time along with the cell population increase. The endophytic infection of *A. brasilense* into the bulb tissues was also confirmed, although these likely constituted vegetative cells. Moreover, the morphological conversion into the c-form was induced under nitrogen-restricted conditions. Analysis of the biochemical properties of the *A. brasilense* cells during cell conversion revealed that the acetylene reduction activity correlated positively with the PHB accumulation in the cells converting into the c-form under nitrogen-restricted conditions.

## 1. Introduction

Plant growth-promoting bacteria (PGPB) confer beneficial effects on the host plants, including the enhancement of growth and productivity, and in return, PGPB benefit from the nutrients provided by the host plant. The genus *Azospirillum* constitutes a well-known PGPB that promotes the host plant’s growth in association with the plant roots through various biochemical abilities, including biological nitrogen fixation (BNF) and the production of phytohormones such as indole-3-acetic acid (IAA), which stimulate root system proliferation [1,2,3]. Specifically, *Azospirillum* is a Gram-negative, spirillum (vibrio)-shaped motile bacterium that exhibits polar flagella in the liquid medium but is peritrichous in the semi-solid medium, for swimming [4]. Thus, chemotaxis toward chemoattractants such as organic acids, sugars, sugar alcohols, and amino acids, is considered to draw *Azospirillum* to the plant roots [5,6]. 

However, when the *Azospirillum* cells are under stress conditions such as desiccation, ultraviolet radiation, osmotic shock, or nutrient deficiency, the cell form is converted into capsulated cyst-like cells (i.e., c-form cells) that are less motile, with an increase in cell size, compared to vegetative cells [7,8,9]. In addition, the cells aggregate and flocculate, owing to the development of an outer layer coat of polysaccharides [10]. The production of extracellular polysaccharides (EPS) in flocs plays an important role in the infection process in the rhizosphere, which renders it possible for bacteria to colonize the surface of the plant roots [11,12]. Moreover, the accumulation of the poly-β-hydroxybutyrate (PHB) granules in the cell matrix causes the morphological conversion of the *Azospirillum* cells [13,14] and is considered as an alternative carbon and energy source for growth and BNF [3,15,16,17].

BNF is a process to convert molecular dinitrogen (N_2_) in the atmosphere into ammonia (NH_3_) and provides nitrogen for plants [18]. The enzyme nitrogenase is essential for fixing nitrogen and extremely sensitive to the molecular oxygen (O_2_) [14], indicating that nitrogen-fixing microorganisms are required to protect nitrogenase from O_2_ [15,19]. BNF is considered to be carried out by two groups of prokaryote, composed of free-living bacteria such as cyanobacteria and *Azotobacter*, and plant-associated bacteria such as *Azospirillum* (associated with cereal) and *Rhizobium* (associated with legume) [20]. For the plant-associated bacteria, *Azospirillum* is recognized as an epiphyte localized on the root surface of the host plant [2,21,22], and considered to defend nitrogenase against O_2_ by the morphological conversion into the c-form as described above. However, *Rhizobium* fixes nitrogen as bacteroids in the nodules formed in the roots of leguminous plants, and the partial pressure of O_2_ inside the nodules is maintained at lower levels by symbiotic hemoglobin (leghemoglobin) produced by legumes [23].

To date, *Azospirillum* has been used to promote the growth of some crops such as wheat [3,9], barley [22], and maize [24]. Recently, a microbial inoculant containing *Azospirillum* has also been applied to onion, which represents a major crop in Hokkaido, Japan [25]. However, no information has been published regarding the interactions between the *Azospirillum* strains and onion, including plant tissue localization of *Azospirillum* and morphological conversion into the c-form cells throughout the infection process. 

In this study, we focused on investigating the initial interactions of *Azospirillum brasilense* with onion seedlings. First, we constructed the *A. brasilense* cells that were labeled by the introduction of the reporter genes, through which the morphological conversion into the c-form as well as the plant tissue localization of the *A. brasilense* cells in the onion seedlings were investigated in detail. Then, a bacterial culture system to induce the morphologically converted c-form was constructed under nitrogen-restricted conditions, and the biochemical characteristics of the *A. brasilense* cells during the conversion into the c-form were studied. We found that the *A. brasilense* cell conversion on the root and bulb (underground stem) surface began after three days and advanced in a time- and cell number-dependent manner, and that the acetylene reduction activity was proportionate to the PHB accumulation in the converting cells.

## 2. Materials and Methods

### 2.1. Bacterial Strains and Media

*A. brasilense* 1224^T^ was provided by the RIKEN BRC through the National Bio-Resource Project of the Ministry of Education, Culture, Sports, Science and Technology, Japan. This strain was grown at 30 °C in the minimal medium (MMAB) [26]. The *Escherichia coli* JM109 and MM294 harboring helper plasmid (pRK2013) were grown at 30 °C in the nutrient broth medium (Eiken Chemical Co. Ltd., Tokyo, Japan). Media were supplemented with appropriate antibiotics at the following concentrations as required: Ampicillin (50 µg/mL), streptomycin (25 µg/mL), and kanamycin (30 µg/mL).

### 2.2. Introduction of Reporter Genes into A. brasilense

The DNA fragment (781 bp), including *mCherry*, was digested with *Eco*RI and *Hind*III from the plasmid pmCherry (TaKaRa Bio Inc., Shiga, Japan) and ligated into a broad host range vector, pBBR1MCS-2 [27] at the site of *Eco*RI-*Hind*III to create pBBR1MCS-2::*mCherry*. Then, the pBBR1MCS-2::*mCherry* or another plasmid, pHRGFPGUS, expressing both *gfp* and *gus* reporter genes constitutively under the control of a gentamycin resistant gene promoter [28], was introduced into the *A. brasilense* cells to construct the mCherry or GFP/GUS-labeled cells by triparental mating, according to Ditta et al. [29]. Briefly, each culture of *E. coli* JM109 (donor), *E. coli* MM294 (helper), and *A. brasilense* (recipient) grown at 30 °C, 150 rpm for 24 h, was centrifuged at 4400× *g* and cell pellets were suspended in a phosphate-buffered saline (PBS) [30]. Resuspension of each cell pellet into PBS after centrifugation was repeated twice and the final cell suspension was prepared in a 500 µL PBS. Two *E. coli* strains (100 µL each) and *A. brasilense* (200 µL) were mixed together, and after centrifugation, the mixed cell pellets were suspended in a 200 µL PBS. From this, 50 µL was taken and mating was conducted at 30 °C for 2–3 days on a membrane filter (mixed cellulose ester; pore size of 0.45 µm, Advantec Co., Tokyo, Japan) placed on the nutrient agar medium. Cell suspensions were spread on MMAB agar plates containing streptomycin, ampicillin, and kanamycin. 

### 2.3. Plasmid Stability

The frozen stocks of the *A. brasilense* cells harboring pHRGFPGUS or pBBR1MCS-2::*mCherry* were recovered on MMAB agar plates, including streptomycin, ampicillin, and kanamycin, at 30 °C for 24 h. The colonies formed were cultured in the MMAB liquid medium without antibiotics at 30 °C for 24 h and then diluted at 1/100 into the same fresh medium. Cultures were allowed to grow for 24 h and then diluted again. This was repeated until 120 h after the first incubation. The ratio (%) of the antibiotics-resistant cell number was determined by the plate dilution method every 24 h following the incubation [31].

### 2.4. Evaluation of Plant Growth Promotion by A. brasilense

The onion seeds (*Allium cepa* L, cultivar kitakogane) were purchased from Takii Seeds Co., Ltd. (Kyoto, Japan). After the seeds were washed by the ultrasonic treatment (200 W; US-105, SND Co., Ltd., Nagano, Japan) for 1 min to remove coating materials, they were sterilized with 90% (*v/v*) ethanol for 1 min, 2% (*v/v*) of sodium hypochlorite with 0.02% (*v/v*) of Tween 20 for 10 min, and then rinsed with sterilized distilled water (three times). The sterilized seeds were left in sterilized water with mild shaking for 20 min, then sown on plant agar plates composed of a nitrogen-free medium (0.7 g K_2_SO_4_, 0.2 g CaSO_4_∙2H_2_O, 0.2 g MgSO_4_∙7H_2_O, 0.2 g Ca_3_(PO_4_)_2_, 0.2 g FePO_4_∙4H_2_O, 0.5 g K_2_HPO_4_, and 7 g agar for plant culture medium per L) and grown in a growth chamber (BiOTRON; NK system, Osaka, Japan) under cycles of 12 h light/dark at 23.5 °C for 10 days. Then, the seedlings were transferred to plant cultivation tubes (150 × 25 mm dia., IWAKI_CTE33_, AGC Techno Glass Co., Shizuoka, Japan) containing a plant agar medium as described above and inoculated with 100 µL of approximately 10^7^ colony forming units/mL *A. brasilense* cells per seedling. All seedlings were grown for four weeks in a growth chamber under the same conditions as described above. Seedlings were weighed before and after drying at 60 °C for seven days to obtain the fresh and dry weights, respectively.

### 2.5. Plant Tissue Localization and Morphological Conversion of A. brasilense in the Onion Seedlings

Ten-day-old seedlings grown as described in Section 2.4 were transferred to fresh plant agar plates and inoculated with the GFP/GUS-labeled *A. brasilense* cells (10^6^ cells per seedling). Plant samples were taken at zero, three, seven, and twenty eight days after the inoculation, and the labeled cells localized in seedlings were stained by immersing the plant samples in a GUS-staining solution (20 mL of 125 mM sodium phosphate buffer (pH 7.0), 100 µL of 0.5 M Na_2_EDTA (pH 8.0), 100 µL of 10% (*w/v*) sodium dodecyl sulfate (SDS), 100 µL of 2% (*w/v*) X-Gluc (5-bromo-4-chloro-3-indolyl-β-d-glucuronide) cyclohexylammonium salt, and 28.8 mL of distilled water) with continuous deaeration in a desiccator connected to a vacuum pump for 30 min, and then incubating at 30 °C for two days. Microscopic observation of the localized *A. brasilense* cells in the seedlings was carried out using a stereomicroscope (SZX16, Olympus Co., Tokyo, Japan). In parallel, the labeled cells localized in the seedlings were also observed under an inverted fluorescence microscope (BZ-X700, Keyence Co., Osaka, Japan) to detect the morphological conversion with the PHB granules inside the cells. Particularly, for the observation of cells localized inside the plant tissues of onion bulbs, the mCherry-labeled cells were used instead of the GFP/GUS-labeled cells to reduce the self-fluorescence emitted by the plant tissues. Agar (5% (*w/v*))-embedded sections of the bulb were sliced using a microslicer (DTK1000 ZERO 1, Dosaka EM Co., Ltd., Kyoto, Japan) and the localized cells inside the bulb were observed under an inverted fluorescence microscope as described above.

### 2.6. Morphological Observation of A. brasilense by Scanning Electron Microscopy (SEM)

The *A. brasilense* cells localized on the surface and inside the bulb of onion plants were observed by GUS staining, followed by SEM. Briefly, the GFP/GUS-labeled cells localized in seedlings (28 days after the inoculation) were stained with the staining solution as described in Section 2.5 and a section of the bulb was sliced using a microslicer to obtain a thickness of approximately 150 µm. The surface and sliced samples were immersed in 2% (*w/v*) of glutaraldehyde for 2 h, washed three times with 0.1 M of phosphate buffer for 15 min, treated with 1% (*w/v*) of osmic acid for 2 h, and washed again as described above. Samples were treated with a series of 50%, 60%, 70%, 80%, 90%, and 99.5% (*v/v*) ethanol for 15 min, and immersed three times in tert-butyl alcohol at 40 °C for 15 min. Then, the samples were lyophilized with a t-BuOH freeze dryer, VFD-21S (VD, Ibaraki, Japan), coated with gold using an MSP-mini magnetron sputterer (VD) and observed with a TM3030 Miniscope (Hitachi-Hi-Tech Co., Tokyo, Japan). 

### 2.7. Detection of Intracellular PHB Granules by Nile Blue Staining

Nile blue staining of the PHB granules was conducted according to the method by Ostel and Holt [32]. Briefly, bacterial cells were heat-fixed on a glass slide and stained with 1% (*w/v*) of the Nile blue A solution at 55 °C for 10 min. Then, the excess stain solution was removed by rinsing with tap water followed by 8% (*v/v*) of an aqueous acetic acid.

### 2.8. Biochemical Analyses of A. brasilense under Nitrogen-restricted Conditions

Production of PHB was quantified as a crotonic acid by high performance liquid chromatography (HPLC) [33]. Briefly, the *A. brasilense* cells were grown at 30 °C, 150 rpm in 50 mL MMAB and its nitrogen-restricted medium (5 mM NH_4_Cl) for three and seven days. The cells were collected by centrifugation at 6300× *g* for 10 min, and after washing with 1 mL PBS, left at 60 °C until the cell weight no longer changed. After measuring the dry weight of the cells, the dry cells were treated with 1 mL of concentrated H_2_SO_4_ at 90 °C for 30 min and cooled down in ice. Then, 4 mL of the phosphoric acid (0.1% *v/v*) was added and mixed, and after dilution, the samples were filtered through a membrane filter (polytetrafluoroethylene; pore size 0.45 µm, Advantec Co., Tokyo, Japan), and a portion of 5 µL was subjected to HPLC (DP8020; Tosoh Co., Tokyo, Japan) equipped with a UV monitor (UV-8020, Tosoh Co.) and a column of TSK-GEL ODS100V (Tosoh Co.) at 40 °C under a flow rate of 1 mL of the phosphoric acid (0.1% *v/v*) per min. Amounts of the crotonic acid production were measured using the correlogram drawn by the absorbance value (210 nm), which was obtained using a known value of the crotonic acid, and converted into the PHB production [33].

The production of IAA was quantified by HPLC [34]. In brief, the *A. brasilense* cells were grown at 30 °C, 150 rpm in 50 mL MMAB containing 0.5 mM l-tryptophan and its nitrogen-restricted medium (5 mM NH_4_Cl) containing 0.5 mM l-tryptophan for three and seven days. The cells were collected by centrifugation and the dry weight of the cells was measured as described above. In parallel, the pH of the supernatant was adjusted to 2.5 by the addition of 1 N HCl and IAA was extracted twice with the same volume (50 mL) of ethyl acetate. Then, the ethyl acetate fraction was evaporated at 35 °C under reduced pressure and the residue was dissolved in 5 mL methanol. Next, 10 µL of the sample was subjected to HPLC as described above, except that 0.1% (*v/v*) of the phosphoric acid in acetonitrile/water (40:60) was used as an eluent. The IAA production was measured using a standard curve drawn by the absorbance value (254 nm) obtained with a known value of IAA.

The nitrogen-fixing ability was measured by determining the acetylene reduction activity (ARA) [35]. Briefly, the *A. brasilense* cells were grown at 30 °C, 150 rpm in 50 mL MMAB and its nitrogen-restricted medium (5 mM NH_4_Cl) for three and seven days. The cells were collected by centrifugation and resuspended in a fresh nitrogen-free MMAB medium. Then, the culture was sealed in a 300 mL flask containing 10% (*v/v*) of acetylene and incubated at 30 °C, 150 rpm for 2 h. A portion of the gas (1 mL) was withdrawn from the sealed flask and applied to a gas chromatograph (GC-8A, Shimadzu Co., Kyoto, Japan) equipped with a column of Porapak N (50/80 mesh) (Waters, Milford, CT, USA). In parallel, cell protein contents were measured to calculate the specific activity; i.e., cells in the culture were lysed with 0.1 M NaOH at room temperature for 30 min and the total cell protein was determined using the Quick Start^TM^ Bradford protein assay (Bio-Rad, Hercules, CA, USA). 

### 2.9. Statistical Analysis

The statistical analysis was performed using the SPSS Statistics for Windows v.22.0 (IBM, Armonk, NY, USA). Data were subjected to the analysis of variance (ANOVA) or the Mann-Whitney *U* test. A post-hoc comparison of mean values among treatments was performed using Tukey’s honestly significant difference (HSD) test at the 5% confidence level. All experiments were performed with a minimum of three replicates for each treatment. 

## 3. Results

### 3.1. Effect of A. brasilense on the Growth of Onion Seedlings

**Figure 1:**
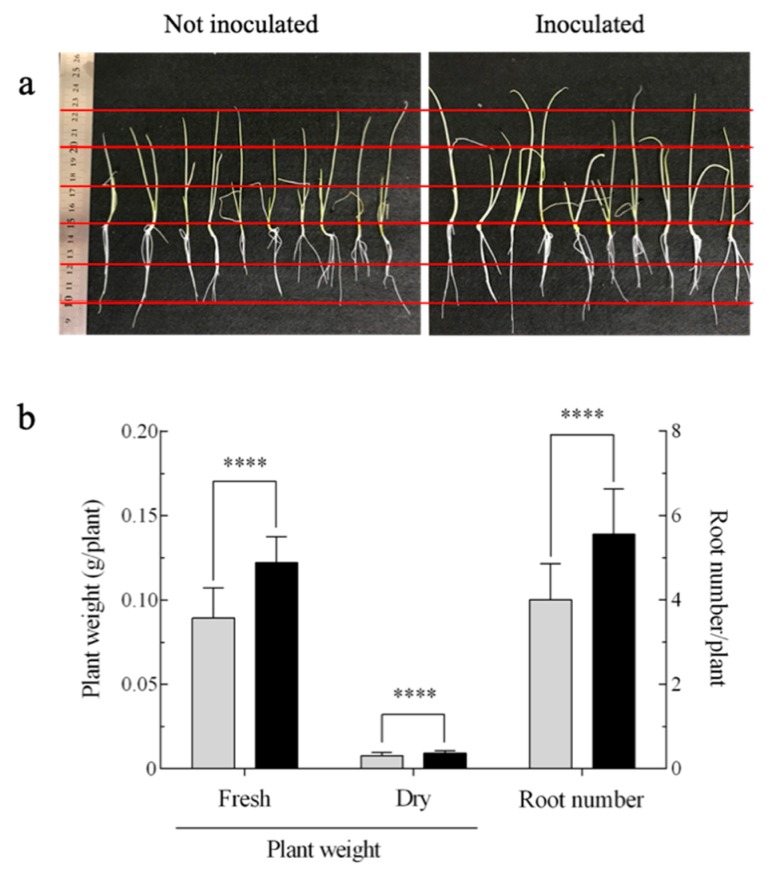
Effect of *Azospirillum brasilense* 1224^T^ harboring the plasmid pHRGFPGUS on the growth of onion seedlings at 28 days post-inoculation. (**a**) Photographs of onion seedlings (distance between each red line is 2.5 cm); (**b**) plant weights (fresh and dry) and root numbers are shown as the means ± standard deviation (SD) calculated from 36 replicates; mean values were compared using the Mann-Whitney *U* test (**** *p* < 0.001). Bars: 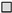, not inoculated; ■, inoculated.

The *A. brasilense* harboring pHRGFPGUS was evaluated for its growth-promoting effect on onion seedlings at 28 days, following inoculation (Figure 1 and Figure S1). The results showed that the *A. brasilense* cells tended to promote the length of plants by approximately 20% compared with the uninoculated control (Figure 1a). In particular, the plant weight and root number were significantly increased with the inoculation of the *A. brasilense* cells; i.e., the former was increased by approximately 37% (*p* ≤ 0.001) and 18% (*p ≤* 0.001) in terms of fresh and dry weights, respectively, and the latter was increased by approximately 37% (*p* ≤ 0.001) compared with the uninoculated control (Figure 1b and Figure S1). 

### 3.2. Stability of Plasmids pHRGFPGUS and pBBR1MCS-2::mCherry in A. brasilense 

As antibiotics used to maintain the plasmids expressing reporter genes are toxic to the host plant, the inoculation test was conducted in the absence of antibiotics. Thus, the maintenance of the plasmids in the *A. brasilense* cells without antibiotics was evaluated. The *A. brasilense* cells harboring pHRGFPGUS or pBBR1MCS-2::*mCherry* were grown without antibiotics five consecutive times for 120 h of incubation (which corresponds to approximately 35 generations) and the plasmid maintenance ratio (%) was monitored (Figure S2). The results showed that approximately 90% of the bacterial cells maintained the plasmid at the end of the incubation (maintenance ratio (%) of 88.1 and 91.7 for pHRGFPGUS and pBBR1MCS-2::*mCherry*, respectively), indicating that both plasmids are stable in the *A. brasilense* cells.

### 3.3. Plant Tissue Localization of A. brasilense in the Onion Seedlings

**Figure 2:**
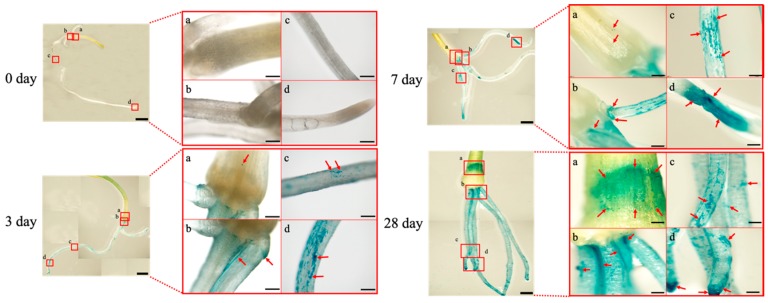
Plant tissue localization of *Azospirillum brasilense* 1224^T^ harboring the plasmid pHRGFPGUS in the onion seedlings. The cells localized in the seedlings at zero, three, seven, and 28 days post-inoculation were observed using a stereomicroscope following GUS-staining. (**a**) Bulb (underground stem), (**b**) base of the root, (**c**) middle of the root, (**d**) tip of the root. Boxes show the enlarged region of the insets, and arrows indicate the localization of the cells. Scale bars: Left panels, 1 mm; right panels, 200 μm.

Figure 2 shows the plant tissue localization of *A. brasilense* upon initial interaction with the onion seedlings. The *A. brasilense* cells mostly colonized in the underground part of the plant such as the roots and bulb by three days post-inoculation as demonstrated in GUS-stained plant tissues. In particular, the cells exhibited efficient colonization in the base, middle, and tip of the roots until seven days post-inoculation (panels b, c, and d for three and seven days post-inoculation, Figure 2). At 28 days post-inoculation, colonization occurred throughout the roots and the cell population was considerably increased compared with that at seven days post-inoculation. Notably, the cell population in the bulb was dramatically increased compared with that at seven days post-inoculation (panel a for seven and 28 days post-inoculation, Figure 2), and the cell density was comparable with that in the base of the roots (panels a and b for 28 days post-inoculation, Figure 2). 

**Figure 3:**
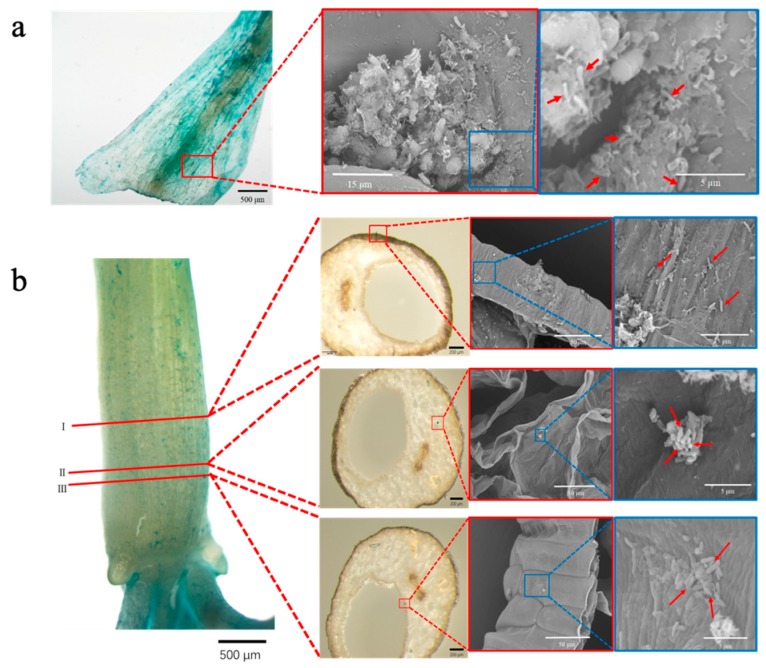
Localization of *Azospirillum brasilense* 1224^T^ harboring the plasmid pHRGFPGUS on the surface and inside the bulb (underground stem). The cells localized on the surface (**a**) and inside (**b**) the bulb at 28 days post-inoculation were detected using GUS-staining (albeit the GUS-staining solution was without SDS and Na_2_EDTA) (left panels) and then observed using SEM (right panels). The transverse bulb sections were prepared at sites I, II, and III as shown in (**b**); red boxes show the enlarged GUS-stained region of the insets, and blue boxes show the magnification of cells in the red boxes. Arrows indicate the localization of the cells.

In addition, the colonization profiles on the surface of and inside the bulb at 28 days post-inoculation were observed by SEM (Figure 3a,b). The results showed that the *A. brasilense* cells spread over the surface, although some cells stuck together to form clumps and localized around the crack (Figure 3a). In addition, the transverse bulb sections were obtained and the internal infection of the *A. brasilense* cells into the bulb tissues was assessed using GUS-staining and SEM (Figure 3b). Notably, the *A. brasilense* cells were also found to colonize in plant tissues inside the bulb (Figure 3b, sections I, II, and III; the cells appeared to be connected to each other with a thread-like material that was visualized by SEM as EPS (section I) [21], colonized between plant tissues in vascular bundles (section II), and localized between bulb scales to form a cell clump (section III).

### 3.4. Morphological Conversion of A. brasilense Cells Localized in the Onion Seedlings

**Figure 4:**
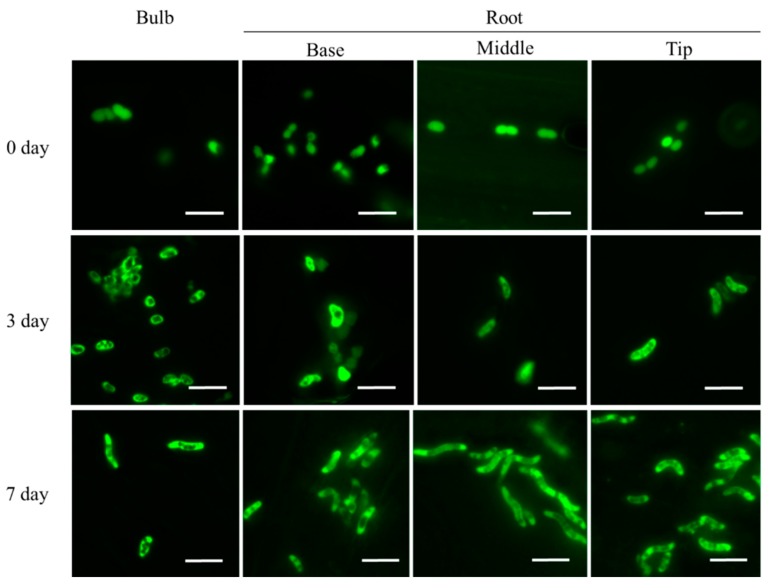
Fluorescence observation of *Azospirillum brasilense* 1224^T^ harboring the plasmid pHRGFPGUS localized on the surface of the bulb (underground stem) and roots of the onion seedlings following inoculation. The cells localized on the surface of the bulb and roots (base, middle, and tip) of the seedlings at zero, three, and seven days post-inoculation were observed using an inverted fluorescence microscope. Scale bars: 5 µm.

Figure 4 shows the fluorescence image of the GFP-labeled *A. brasilense* cells localized on the surface of the bulb and roots (base, middle, and tip) of the onion seedlings until seven days post-inoculation. Notably, the PHB granules were detected as “black holes” in the GFP-labeled *A. brasilense* cells (harboring the plasmid pHRGFPGUS) under fluorescence, indicating that the morphological conversion from vegetative to c-form cells, which is associated with the production of PHB granules inside the cells, could be monitored continuously with time following the inoculation. Results obtained by fluorescence imaging indicated that the cells contained no PHB granules at zero days post-inoculation (Figure 4, zero day). However, at three days post-inoculation, almost all of the cells showed intracellular PHB granules and the number of cells containing PHB granules tended to increase with time (Figure 4, three and seven days). 

Table 1 shows the length of cells (μm) localized on the surface of the bulb and roots (base, middle, and tip) of the onion seedlings at three and seven days post-inoculation. The results indicated that the cell length was approximately 1.66 μm to 1.87 μm at zero days post-inoculation. However, the cell length at both the bulb and roots was significantly elongated with time, and the values reached approximately 4.67 μm (bulb), 4.24 μm (base of the roots), 6.61 μm (middle of the roots), and 4.55 μm (tip of the roots) at seven days post-inoculation, which corresponded to approximately 2.7, 2.4, 3.5, and 2.7-fold, respectively, of those at zero days post-inoculation (Table 1). These results demonstrated that vegetative cells localized on the surface of the bulb and roots of the onion seedlings start converting to the c-form cells at three days post-inoculation, although the cell length (conversion rate of cells) varied depending on the localization in the onion seedlings, even within the roots (Table 1).

**Figure 5:**
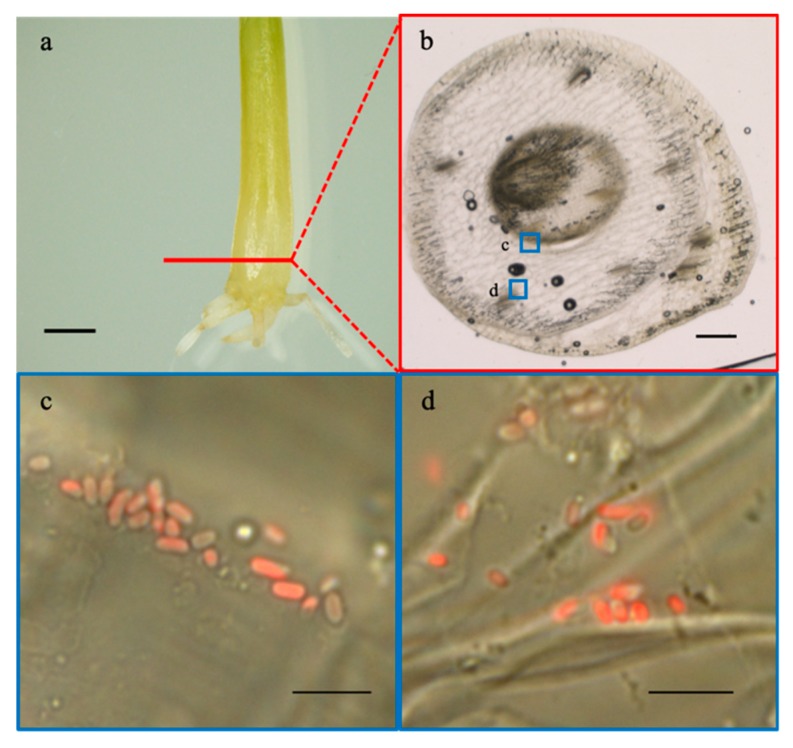
Fluorescence observation of *Azospirillum brasilense* 1224^T^ harboring the plasmid pBBR1MCS-2::*mCherry* inside the bulb (underground stem) tissues of the onion seedlings at 28 days post-inoculation. The transverse bulb section (**b**) was prepared by slicing the bulb at a red line as shown in (**a**), and the cells localized in the section were observed using an inverted fluorescence and optical microscope. Boxes show the enlarged region of the insets. (**a**) Bulb (underground stem); (**b**) transverse bulb section; panels (**c**) and (**d**): Overlay of optical and fluorescence images. Scale bars: (**a**), 4 mm; (**b**), 500 µm; (**c**) and (**d**), 5 µm.

The morphological conversion of the *A. brasilense* cells inside the bulb tissues at 28 days post-inoculation was assessed using the mCherry-labeled cells (harboring the plasmid pBBR1MCS-2::*mCherry*), in order to overcome autofluorescence from the transverse bulb sections (Figure 5). Colonization of the cells was found between the bulb scales (Figure 5, panel c) and plant tissues in vascular bundles (Figure 5, panel d), respectively. However, no cells exhibiting “black holes” were detected under fluorescence (Figure 5, panels c and d), indicating the lack of PHB granules in the cells. In addition, the cell length (μm) was comparable to that at zero days post-inoculation in the onion seedlings (1.66 μm to 1.87 μm) (Figure 4, Table 1). These results indicated that the cells inside the bulb tissues were vegetative and not converted to the c-form.

### 3.5. Biochemical Characteristics of Morphologically Converted A. brasilense Cells

**Figure 6:**
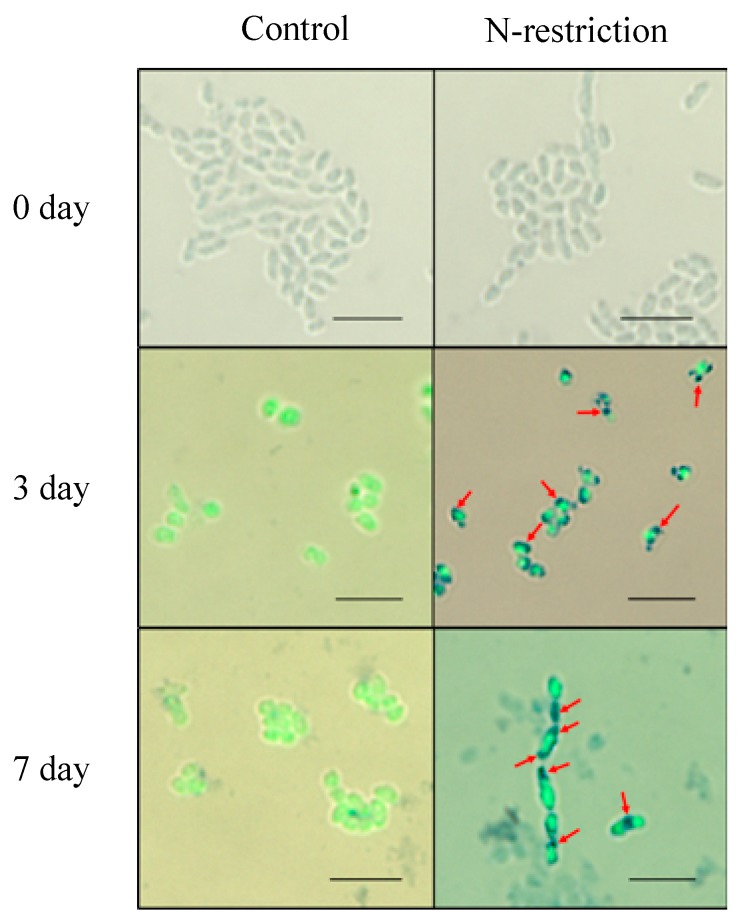
Morphological conversion of *Azospirillum brasilense* 1224^T^ harboring the plasmid pHRGFPGUS into the c-form during incubation in the nitrogen-restricted MMAB medium. The cells were grown at 30 °C for three and seven days and were stained with Nile blue A. Photos show the overlay of optical and fluorescence images. Control (left panels): Cells grown in the MMAB medium; N-restriction (right panels): Cells grown in the nitrogen-restricted MMAB medium. Arrows indicate the PHB granules stained with Nile blue A. Scale bars: 5 µm.

**Figure 7:**
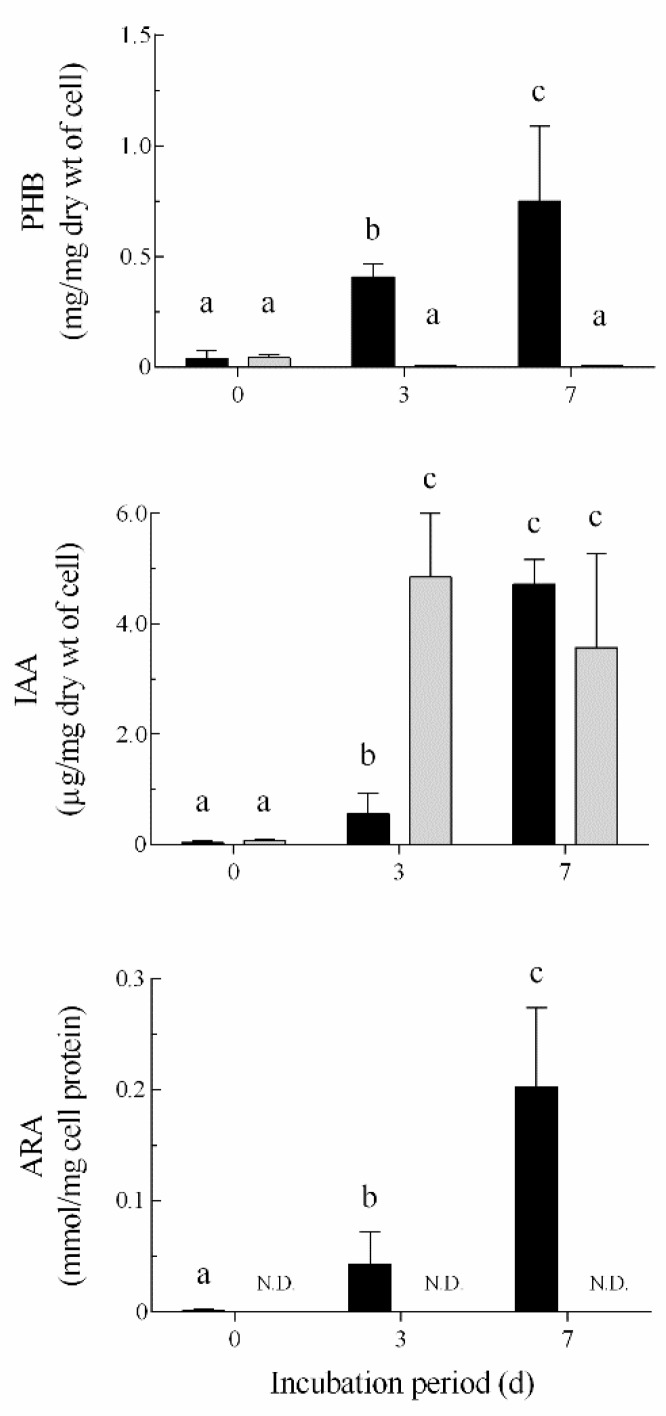
Biochemical analyses of *Azospirillum brasilense* 1224^T^ harboring the plasmid pHRGFPGUS converting morphologically into the c-form during incubation in the nitrogen-restricted MMAB medium. The cells were grown at 30 °C for three and seven days in the MMAB (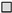) or nitrogen-restricted MMAB medium (■), and the levels of poly-β-hydroxybutyrate (PHB), indole-3-acetic acid (IAA), and acetylene reduction activity (ARA) in the cells were analyzed as described in Materials and Methods. Values are expressed as the means ± standard deviation (SD) of three replicates. Means in the same parameter (PHB, IAA, ARA) with different letters (a, b, c) above each bar are significantly different from each other at *p* < 0.05 by Tukey’s HSD test. N.D., not detected.

To investigate the biochemical characteristics of the morphologically converted *A. brasilense* cells, the morphological conversion of the cells was induced under nitrogen-restricted conditions (i.e., the nitrogen-restricted MMAB medium), and their abilities to produce PHB and IAA as well as the nitrogen-fixing activity, which was based on ARA, were assessed (Figure 6 and Figure 7). Figure 6 shows the morphological conversion of the *A. brasilense* cells during incubation in the nitrogen-restricted MMAB medium. The PHB granules in the cells were stained by Nile blue to make the associated “black holes” clearer. The results showed that almost all of the cells exhibited the presence of the Nile blue-stained PHB granules even at three days post-inoculation, and the cell length continued elongating until seven days post-inoculation (cell length at seven days post-inoculation: Approximately 3.29 μm) (Figure 6). However, under non-nitrogen restricted conditions (i.e., standard MMAB), no cells showed the intracellular Nile blue-stained granules and cell elongation did not occur during incubation (cell length at seven days post-inoculation: Approximately 1.55 μm) (Figure 6). These results indicated that the *A. brasilense* vegetative cells were morphologically converted under nitrogen-restricted conditions. 

Figure 7 shows the production of PHB, IAA, and the ARA ability in the *A. brasilense* cells during incubation in MMAB and nitrogen-restricted MMAB media. The PHB production in the cells in the MMAB medium was very low (< 0.05 mg per mg of the dry cell weight) during incubation. However, the production in the nitrogen-restricted MMAB medium was significantly increased during incubation, with levels reaching approximately 0.75 mg per mg of the dry cell weight, which corresponded to approximately 75% (*w/w*) (*p* ≤ 0.001) of the dry cell weight at seven days post-inoculation (Figure 7). ARA in the cells in the MMAB medium was not detected during incubation. However, in nitrogen-restricted conditions, ARA was conspicuously detected at three days post-inoculation and the level was significantly increased at seven days post-inoculation (approximately four times of that at three days post-inoculation). These results indicated that the enhanced abilities of the PHB production and ARA were caused by the morphological conversion of the *A. brasilense* cells into the c-form, which was induced by nitrogen restriction (Figure 6 and Figure 7). The IAA in the MMAB medium was produced approximately at 4.8 µg per mg of the dry cell weight (*p* ≤ 0.001) at three days post-inoculation and the level tended to decrease at seven days post-inoculation. In comparison, the production in the nitrogen-restricted MMAB medium was significantly lower than that in MMAB (approximately 0.6 µg per mg of the dry cell weight) (*p* ≤ 0.001). However, the level was significantly increased to approximately 4.7 µg per mg of the dry cell weight (*p* ≤ 0.001) at seven days post-inoculation. The results obtained by the IAA analysis showed that the morphologically converted cells also exhibit the IAA production at a level comparable with that in vegetative cells, although the production rate tended to be delayed compared with that of vegetative cells.

## 4. Discussion

In this study, the *A. brasilense* cells exhibited the ability to promote the growth of onion seedlings, as shown particularly by an increase in plant weight and root number, suggesting that the growth of stalk vegetables like onion as well as cereals such as wheat [36] could be stimulated by the inoculation of this bacterial strain (Figure 1 and Figure S1). In the field, a combined inoculation of the *Azospirillum* and AM fungi has been used to improve the growth and yield of the onion [37]. The stable maintenance of the plasmid pHRGFPGUS and pBBR1MCS-2::*mCherry* in *A. brasilense* (Figure S2) prompted us to investigate its plant tissue localization and morphological conversion. 

Plant tissue localization of the *A. brasilense* cells harboring the plasmid pHRGFPGUS was studied by GUS staining and SEM (Figure 2 and Figure 3). The results showed that the *A. brasilense* cells are likely to colonize at the roots (base, middle, and tip) and bulb. In particular, the cell population on the surface of the bulb was markedly increased at 28 days post-inoculation and the density was comparable with that in the base of the roots, suggesting that the plant tissues from the bulb to the base of the roots may play an important role in the initial interactions between the *A. brasilense* cells and onion seedlings (Figure 2, 28 days post-inoculation). The adhesion process of the *Azospirillum* strain to the surface of the plant roots is not yet clear. However, the motility, along with flocculation caused by the production of EPS, have been considered to be important for this process [8,21,38]. It was reported that the motility such as via chemotaxis, enabled the *Azospirillum* strain to move toward the plant roots in which the cells could benefit from the root exudates [13,39]. This suggested that the *Azospirillum* cells are drawn to specific parts of plant tissues such as the roots (base, middle and tip) by root exudates, and that the colonization spreads over the bulb at 28 days post-inoculation (Figure 2).

Recently, the *Azospirillum* cells have been reported to infect the root hairs of barley at 12 days following inoculation as determined, using GUS-staining [22]. However, we confirmed the endophytic infection of the *Azospirillum* cells into plant tissues between the bulb scales to generate characteristic colonization patterns such as cell clumps with EPS production (Figure 3b). To our knowledge, this is the first report of the endophytic infection and colonization of *Azospirillum* in the plant tissues of stalk vegetables such as onions. The results imply that the cells colonized between the bulb scales might have passed through the endodermis and loose vascular tissues from the cell population at a high density on the surface of the bulb. 

Next, the morphological conversion of the *A. brasilense* cells was investigated using the GFP/mCherry-labeled cells under fluorescence (Figure 4 and Figure 5). The morphological conversion of *Azospirillum* from the vegetative to c-form cells is known to be associated with the elongation of cells caused by the formation of intracellular PHB granules, with the transmission electron microscopy being used to identify the c-form cells [10,40]. However, we found that the PHB granules in the *Azospirillum* cells could be readily detected as “black holes” using the GFP/mCherry-labeled cells under fluorescence. Thus, this feature offered a simple and quick method to monitor the morphological conversion of the *Azospirillum* cells from the vegetative to c-form cells (Figure 4). In addition, the mCherry-labeled cells were used to distinguish bacterial cells from plant tissues such as the transverse section of the bulb upon the removal of a strong auto-fluorescence (Figure 5) [41]. Under stressful conditions such as nitrogen deficiency, the morphological conversion of *Azospirillum* into the c-form cells was considered to be necessary for its survival, with this conversion being reported to reach completion at 10 days after the inoculation with wheat, according to cellular morphology based on X-Gal-staining and β-galactosidase activities [8,9]. In the present study, however, we reported that the majority of the *Azospirillum* cells localized on the surface of the bulb and roots of the onion seedlings had begun converting into the c-form at three days post-inoculation and the conversion advanced until at least seven days post-inoculation under nitrogen-restricted conditions (Figure 4, Table 1). 

To study the biochemical properties of the *Azospirillum* cells during the morphological conversion into the c-form, a bacterial culture system to induce the morphological conversion was successfully constructed using the nitrogen-restricted MMAB medium. The results showed that the PHB accumulation and the increase in ARA occurred in parallel with the morphological conversion into the c-form cells (Figure 6 and Figure 7). As the morphological conversion into the c-form cells is considered to associate with the PHB accumulation in the cells and the protection of nitrogenase from oxygen, the results suggested that ARA correlates positively with the accumulation of PHB in the cells during incubation (Figure 7). Moreover, PHB, which is considered an energy source under stressful conditions, has been reported to also affect the nitrogen-fixing ability [2,42]. The *Azospirillum* cells localized and morphologically converted into the c-form on the surface of the base, middle, and tip of the roots as well as on the bulb (Figure 4). In particular, the morphologically converted cells at the middle of the roots exhibited the most elongated shape caused by the PHB accumulation at seven days post-inoculation, implying a higher ability to fix nitrogen (Table 1). It is considered that the middle roots constitute the maturation zone of the roots and root hairs in this zone obtain nutrients from the rhizosphere environment [43]. Conversely, the *Azospirillum* cells localized inside plant tissues in the bulb appeared to be vegetative cells, suggesting that they do not have the ability to fix nitrogen (Figure 5).

The IAA production of PGPB is considered to be important for plant growth promotion, with tryptophan, a precursor for the IAA biosynthesis, being released from the host plant [16,44]. In addition, the release of tryptophan is stimulated by the plant growth promotion caused by the IAA-mediated production of PGPB, indicating a closed loop system between the IAA production and tryptophan release [17,45]. The results shown in Figure 7 indicate that the morphologically converted c-form cells along with the vegetative cells of *Azospirillum* likely have the ability to produce IAA. However, the IAA production in the morphologically converted *Azospirillum* cells was delayed compared with that in the vegetative cells (Figure 7). It appeared that the PHB synthesis is not associated with the IAA biosynthesis in the c-form cells [46]. Further studies are needed to clarify why the IAA production was delayed in the process of morphological conversion into the c-form.

## 5. Conclusions

Our results indicated that *A. brasilense* could colonize at the bulb as well as the roots of a stalk vegetable, onion, and promote the plant growth. Particularly, the endophytic colonization of the *A. brasilense* cells inside plant tissues was demonstrated and this is the first report for stalk vegetables such as onion. Moreover, the surface-colonized *A. brasilense* cells were found to begin converting into the c-form at three days following inoculation in association with intracellular PHB accumulation, which correlated positively with the increase in ARA (i.e., nitrogen-fixing ability). Further studies are necessary to clarify the process of the endophytic infection and colonization of the *Azospirillum* cells into plant tissues of stalk vegetables such as onion. These findings will facilitate the application and optimization of the *Azospirillum* strains for the agricultural production of stalk vegetables including onion.

## Figures and Tables

**Table 1:** Cell length o*f Azospirillum brasilense* 1224^T^ harboring the plasmid pHRGFPGUS localized on the surface of the bulb and roots of the onion seedlings following inoculation.

Plant Tissue	Days after Inoculation		
	0	3	7
Bulb	1.73 ± 0.40 ^ab^	2.17 ± 0.52 ^bc^	4.67 ± 1.14 ^f^
Roots			
Base	1.75 ± 0.34 ^ab^	2.75 ± 0.43 ^d^	4.24 ± 1.09 ^f^
Middle	1.87 ± 0.37 ^ab^	2.38 ± 0.47 ^cd^	6.61 ± 1.57 ^g^
Tip	1.66 ± 0.31 ^a^	3.40 ± 0.53 ^e^	4.55 ± 1.20 ^f^

Values (µm) represent the means ± standard deviation (SD) calculated from 50 cells. A two-way ANOVA was performed to compare the significance of differences between the means. Mean values with common superscript letters are not significantly different from each other (*p* ≤ 0.05) by Tukey’s HSD test.

## Supplementary Materials

The following are available online at https://www.mdpi.com/2076-2607/7/9/275/s1. Figure S1: Effect of *Azospirillum brasilense* 1224^T^ harboring the plasmid pHRGFPGUS on the growth of onion seedlings at 28 days post-inoculation. Figure S2: Maintenance of plasmids pHRGFPGUS and pBBR1MCS-2::*mCherry* in *Azospirillum brasilense* 1224^T^ in the absence of antibiotics.

## References

[1] Herschkovitz Y., Lerner A., Davidov Y., Rothballer M., Hartmann A., Okon Y., Jurkevitch E. (2005). Inoculation with the plant-growth-promoting rhizobacterium *Azospirillum brasilense* causes little disturbance in the rhizosphere and rhizoplane of maize (*Zea mays*). Microb. Ecol..

[2] Steenhoudt O., Vanderleyden J. (2000). *Azospirillum*, a free-living nitrogen-fixing bacterium closely associated with grasses: genetic, biochemical and ecological aspects. FEMS Microbiol. Rev..

[3] Spaepen S., Dobbelaere S., Croonenborghs A., Vanderleyden J. (2008). Effects of *Azospirillum brasilense* indole-3-acetic acid production on inoculated wheat plants. Plant Soil.

[4] Hall P.G., Krieg N.R. (1983). Swarming of *Azospirillum brasilense* on solid media. Can. J. Microbiol..

[5] Okon Y., Cakmakci L., Nur I., Chet I. (1980). Aerotaxis and chemotaxis of *Azospirillum brasilense*: a note. Microb. Ecol..

[6] Alexandre G., Greer S.E., Zhulin I.B. (2000). Energy taxis is the dominant behavior in *Azospirillum brasilense*. J. Bacteriol..

[7] Okon Y., Itzigsohn R. (1992). Poly-*β*-hydroxybutyrate metabolism in *Azospirillum brasilense* and the ecological role of PHB in the rhizosphere. FEMS Microbiol. Lett..

[8] Pereg Gerk L., Gilchrist K., Kennedy I.R. (2000). Mutants with enhanced nitrogenase activity in hydroponic *Azospirillum brasilense* -wheat associations. Appl. Environ. Microbiol..

[9] Katupitiya S., Millet J., Vesk M., Viccars L., Zeman A., Lidong Z., Elmerich C., Kennedy I.R. (1995). A mutant of *Azospirillum brasilense* Sp7 impaired in flocculation with a modified colonization pattern and superior nitrogen fixation in association with wheat. Appl. Environ. Microbiol..

[10] Sadasivan L., Neyra C.A. (1985). Flocculation in *Azospirillum brasilense* and *Azospirillum lipoferum*: exopolysaccharides and cyst formation. J. Bacteriol..

[11] Burdman S., Jurkevitch E., Schwartsburd B., Hampel M., Okon Y. (1998). Aggregation in *Azospirillum brasilens*e: effects of chemical and physical factors and involvement of extracellular components. Microbiology.

[12] Burdman S., Okon Y., Jurkevitch E. (2000). Surface characteristics of *Azospirillum brasilense* in relation to cell aggregation and attachment to plant roots. Crit. Rev. Microbiol..

[13] Compant S., Clément C., Sessitsch A. (2010). Plant growth-promoting bacteria in the rhizo-and endosphere of plants: their role, colonization, mechanisms involved and prospects for utilization. Soil Biol. Biochem..

[14] Bible A.N., Khalsa-Moyers G.K., Mukherjee T., Green C.S., Mishra P., Purcell A., Aksenova A., Hurst G.B., Alexandre G. (2015). Metabolic adaptations of *Azospirillum brasilense* to oxygen stress by cell-to-cell clumping and flocculation. Appl. Environ. Microbiol..

[15] Nur I., Okon Y., Henis Y. (1982). Effect of dissolved oxygen tension on production of carotenoids, poly-*β*-hydroxybutyrate, succinate oxidase and superoxide dismutase by *Azospirillum brasilense* Cd grown in continuous culture. J. Gen. Microbiol..

[16] Palacios O.A., Gomez-Anduro G., Bashan Y., de-Bashan L.E. (2016). Tryptophan, thiamine and indole-3-acetic acid exchange between *Chlorella sorokiniana* and the plant growth-promoting bacterium *Azospirillum brasilense*. FEMS Microbiol. Ecol..

[17] Kamilova F., Kravchenko L.V., Shaposhnikov A.I., Azarova T., Makarova N., Lugtenberg B. (2006). Organics acids, sugars, and L-tryptophane in exudates of vegetables growing on stonewool and their effects on activities of rhizosphere bacteria. Mol. Plant Microbe Interact..

[18] Rascio N., Rocca N.L., Fath B.D. (2018). Biological nitrogen fixation. Encyclopedia of Ecology.

[19] Oke V., Long S.R. (1999). Bacteroid formation in the *Rhizobium*–legume symbiosis. Curr. Opin. Biotechnol..

[20] Wagner S.C. (2011). Biological nitrogen fixation. Nat. Educ. Knowl..

[21] Pereg Gerk L., Paquelin A., Gounon P., Kennedy I.R., Elmerich C. (1998). A transcriptional regulator of the LuxR-UhpA family, FlcA, controls flocculation and wheat root surface colonization by *Azospirillum brasilense* Sp7. Mol. Plant Microbe Interact..

[22] Santos A.R.S., Etto R.M., Furmam R.W., Freitas D.L., Santos K.F.D.N., Souza E.M., Pedrosa F.O., Ayub R.A., Steffens M.B.R., Galvão C.W. (2017). Labeled *Azospirillum brasilense* wild type and excretion-ammonium strains in association with barley roots. Plant Physiol. Biochemi..

[23] Tajepkema J.D., Yocum C.S. (1974). Measurement of oxygen partial pressure within soybean nodules by oxygen microelectrodes. Planta..

[24] Okon Y., Kapulnik Y. (1986). Development and function of *Azospirillum*- inoculated roots. Plant Soil.

[25] Ministry of Agriculture, Forestry and Fisheries. http://www.maff.go.jp/hokkaido/toukei/index.html.

[26] Vanstockem M., Michiels K., Vanderleyden J., Van Gool A.P. (1987). Transposon mutagenesis of *Azospirillum brasilense* and *Azospirillum lipoferum*: physical analysis of Tn*5* and Tn*5*-mob insertion mutants. Appl. Environ. Microbiol..

[27] Kovach M.E., Elzer P.H., Hill D.S., Robertson G.T., Farris M.A., Roop R.M., Peterson K.M. (1995). Four new derivatives of the broad-host-range cloning vector pBBR1MCS, carrying different antibiotic-resistance cassettes. Gene.

[28] Ramos H.J.O., Roncato-Maccari L.D., Souza E.M., Soares-Ramos J.R., Hungria M., Pedrosa F.O. (2002). Monitoring *Azospirillum*-wheat interactions using the *gfp* and *gusA* genes constitutively expressed from a new broad-host range vector. J. Biotechnol..

[29] Ditta G., Stanfield S., Corbin D., Helinski D. (1980). Broad host range DNA cloning system for Gram-negative bacteria: Construction of a gene bank of *Rhizobium meliloti*. Proc. Natl. Acad. Sci. USA.

[30] Bashan Y., Mitiku G., Whitmoyer R.E., Levanony H. (1991). Evidence that fibrillar anchoring is essential for *Azospirillum brasilense* Cd attachment to sand. Plant Soil.

[31] Gage D.J., Bobo T., Long S.R. (1996). Use of green fluorescent protein to visualize the early events of symbiosis between *Rhizobium meliloti* and *alfalfa* (*Medicago sativa*). J. Bacteriol..

[32] Ostle A.G., Holt J.G. (1982). Nile Blue A as a fluorescent stain for poly-3- hydroxybutyrate. Appl. Environ. Microbiol..

[33] Karr D.B., Waters J.K., Emerich D.W. (1983). Analysis of poly-3-hydroxybutyrate in *Rhizobium japonicum* bacteroids by ion-exclusion high-pressure liquid chromatography and UV detection. Appl. Environ. Microbiol..

[34] Perrig D., Boiero M.L., Masciarelli O.A., Penna C., Ruiz O.A., Cassán F.D., Luna M.V. (2007). Plant-growth-promoting compounds produced by two agronomically important strains of *Azospirillum brasilense*, and implications for inoculant formulation. Appl. Microbiol. Biotechnol..

[35] Hartmann A., Fu H.A., Burris R.H. (1988). Influence of amino acids on nitrogen fixation ability and growth of *Azospirillum* spp.. Appl. Environ. Microbiol..

[36] Kaushik R., Saxena A.K., Tilak K.V.B.R. (2000). Selection of Tn5::lacZ mutants isogenic to wild type *Azospirillum brasilense* strains capable of growing at sub-optimal temperature. World J. Microbiol. Biotechnol..

[37] Sridevi S., Ramakrishnan K. (2010). Effects of combined inoculation of AM Fungi and *Azospirillum* on the growth and yield of onion (*Allium cepa* L.). J. Phytol..

[38] Hou X., McMillan M., Coumans J.V.F., Poljak A., Raftery M.J., Pereg L. (2014). Cellular responses during morphological transformation in *Azospirillum brasilense* and its flcA knockout mutant. PLoS One.

[39] Naher K., Miwa H., Okazaki S., Yasuda M. (2018). Effects of different sources of nitrogen on endophytic colonization of rice plants by *Azospirillum* sp. B510. Microb. Environ..

[40] Berg R.H., Tyler M.E., Novick N.J., Vasil V., Vasil I.K. (1980). Biology of *Azospirillum*-sugarcane association: enhancement of nitrogenase activity. Appl. Environ. Microbiol..

[41] Ramirez-Mata A., Pacheco M.R., Moreno S.J., Xiqui-Vazquez M.L., Baca B.E. (2018). Versatile use of *Azospirillum brasilense* strains tagged with *egfp* and *mCherry* genes for the visualization of biofilms associated with wheat roots. Microbiol. Res..

[42] Wang C., Sheng X., Equi R.C., Trainer M.A., Charles T.C., Sobral B.W. (2007). Influence of the poly-3-hydroxybutyrate (PHB) granule-associated proteins (PhaP1 and PhaP2) on PHB accumulation and symbiotic nitrogen fixation in *Sinorhizobium meliloti* Rm1021. J. Bacteriol..

[43] Thies J.E., Grossman J.M., Uphoff M., Ball A.S., Fernandes E., Herren H., Husson O., Laing M., Palm C., Pretty J., Sanchez P., Sanginga N. (2006). The soil habitat and soil ecology. Biological strategies for sustainable soil systems.

[44] Spaepen S., Vanderleyden J., Remans R. (2007). Indole-3-acetic acid in microbial and microorganism-plant signaling. FEMS Microbiol. Rev..

[45] Idris E.E., Iglesias D.J., Talon M., Borriss R. (2007). Tryptophan-dependent production of indole-3-acetic acid (IAA) affects level of plant growth promotion by *Bacillus amyloliquefaciens* FZB42. Mol. Plant Microbe Interact..

[46] Malinich E.A., Bauer C.E. (2018). Transcriptome analysis of *Azospirillum brasilense* vegetative and cyst states reveals large-scale alterations in metabolic and replicative gene expression. Microb. Genom..

