# Supplementary material for: Plant Tissue Localization and Morphological Conversion of Azospirillum brasilense upon Initial Interaction with Allium cepa L
Source: Microorganisms. 2019 Aug 21;7(9):275. doi: 10.3390/microorganisms7090275 (PMC6780411; doi:10.3390/microorganisms7090275)
Supplement: Supplementary file 1 [file microorganisms-07-00275-s001.pdf]

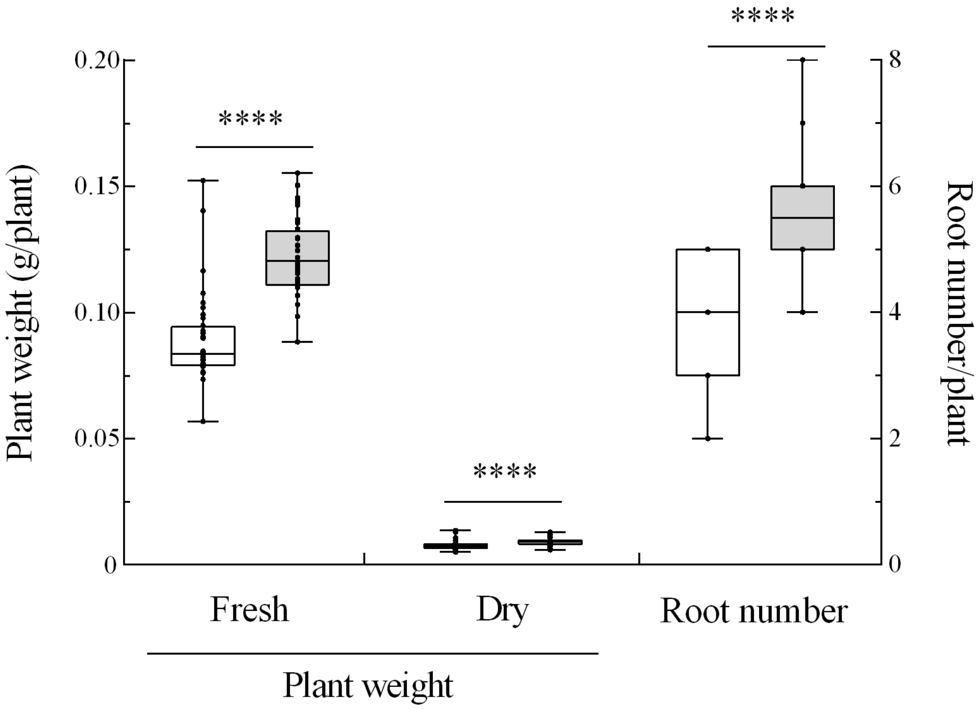

Figure S1. Effect of *Azospirillum brasilense* 1224^T harboring the plasmid pHRGFPGUS on the growth of onion seedlings at 28 days post-inoculation. Plant weights (fresh and dry) and root number are shown in boxplots calculated from 36 replicates. Boxes indicate the median, and first and third quartile; whiskers indicate the minimum and maximum. Mean values were compared using the Mann-Whitney *U* test (****$p < 0.001$). Boxes: □, not inoculated; □, inoculated.

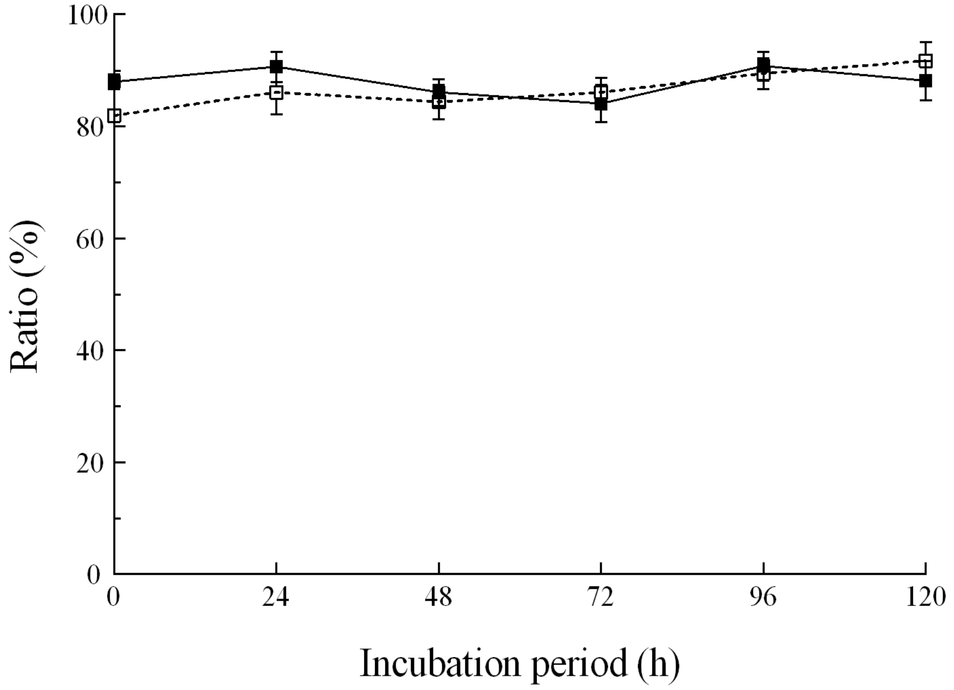

Figure S2. Maintenance of plasmids pHRGFPGUS and pBBR1MCS-2::*mCherry* in *Azospirillum brasilense* 1224^{T} in the absence of antibiotics. The cells were grown without antibiotics five consecutive times (120 h, which corresponds to approximately 35 generations) and the ratio (%) of plasmid maintained in the cells was monitored as described in Materials and Methods. Symbols: ■ and □, maintenance ratio of pHRGFPGUS and pBBR1MCS-2::*mCherry* in *A. brasilense* 1224^{T}, respectively.
